# DC-SIGN of Largemouth Bass (*Micropterus salmoides*) Mediates Immune Functions against *Aeromonas hydrophila* through Collaboration with the TLR Signaling Pathway

**DOI:** 10.3390/ijms25095013

**Published:** 2024-05-03

**Authors:** Mengmeng Huang, Jingwen Liu, Zhenzhen Yuan, Youxing Xu, Yang Guo, Shun Yang, Hui Fei

**Affiliations:** 1 College of Life Sciences and Medicine, Zhejiang Sci-Tech University, Hangzhou 310018, China; mmhuang15@zstu.edu.cn (M.H.);; 2 Zhejiang Provincial Key Laboratory of Silkworm Bioreactor and Biomedicine, Zhejiang Sci-Tech University, Hangzhou 310018, China

**Keywords:** *Micropterus salmoides*, C-type lectins, pattern recognition receptors, DC-SIGN, TLR signaling pathway, phagocytosis

## Abstract

C-type lectins in organisms play an important role in the process of innate immunity. In this study, a C-type lectin belonging to the DC-SIGN class of *Micropterus salmoides* was identified. MsDC-SIGN is classified as a type II transmembrane protein. The extracellular segment of MsDC-SIGN possesses a coiled-coil region and a carbohydrate recognition domain (CRD). The key amino acid motifs of the extracellular CRD of MsDC-SIGN in Ca^2+^-binding site 2 were EPN (Glu-Pro-Asn) and WYD (Trp-Tyr-Asp). MsDC-SIGN-CRD can bind to four pathogen-associated molecular patterns (PAMPs), including lipopolysaccharide (LPS), glucan, peptidoglycan (PGN), and mannan. Moreover, it can also bind to Gram-positive, Gram-negative bacteria, and fungi. Its CRD can agglutinate microbes and displays D-mannose and D-galactose binding specificity. MsDC-SIGN was distributed in seven tissues of the largemouth bass, among which the highest expression was observed in the liver, followed by the spleen and intestine. Additionally, MsDC-SIGN was present on the membrane of *M*. *salmoides* leukocytes, thereby augmenting the phagocytic activity against bacteria. In a subsequent investigation, the expression patterns of the MsDC-SIGN gene and key genes associated with the TLR signaling pathway (TLR4, NF-κB, and IL10) exhibited an up-regulated expression response to the stimulation of *Aeromonas hydrophila*. Furthermore, through RNA interference of MsDC-SIGN, the expression level of the DC-SIGN signaling pathway-related gene (RAF1) and key genes associated with the TLR signaling pathway (TLR4, NF-κB, and IL10) was decreased. Therefore, MsDC-SIGN plays a pivotal role in the immune defense against *A*. *hydrophila* by modulating the TLR signaling pathway.

## 1. Introduction

C-type lectin was originally considered a carbohydrate-binding protein that can recognize carbohydrates depending on Ca^2+^ and contains a carbohydrate recognition domain (CRD) [1,2]. Dendritic cell-specific intercellular-adhesion-molecule-3-grabbing non-integrin (DC-SIGN, or called CD209) belongs to a type II transmembrane protein [3]. The DC-SIGN molecule consists of four domains, including the cytoplasmic domain, the transmembrane domain, the hinge domain, and the extra cytoplasmic domain [4]. Studies have found that DC-SIGN can act on intercellular adhesion molecules (ICAM), such as ICAM-2, ICAM-3, etc., can play a mediating role in the interaction between cells, and can play an important immunomodulatory role after infection and inflammation [5]. In teleost fish, the DC-SIGN molecule was found in zebrafish [6], *Salmo salar* [7], and *Sebastes schlegelii* [8]. In the zebrafish study, it was found that DC-SIGN could respond to the stimulation of *Aeromonas hydrophila*, and its expression was up-regulated in the spleen and gills [6]. SsSIGN in *S*. *salar* was largely expressed in various tissues and responded to bacteria stimulation [7]. After being infected by *Vibrio parahaemolyticus*, the expression of C-type lectin SsCTL4 in the kidney, liver, and spleen was up-regulated over time [8]. Thus, it can recognize pathogens and exert immune response effects.

As a kind of PRR, TLRs can participate in the signal transduction process in cells and play an extremely important role in the synthesis and release of proinflammatory cytokines [9,10]. Continued interactions with bacteria require the co-ordination of multiple PRR signaling pathways that dictate the outcome of microbial colonization [11]. Studies have shown that C-type lectins can collaborate with TLRs to exert immunomodulatory effects through the TLR signaling pathway. For example, the knocking down of Clec2d eliminated the activation of the TLR9-NF-κB signaling pathway in mice with chronic stress [7]. DC-SIGN interacts with *Helicobacter pylori* and is capable of releasing signals to modulate TLR signaling [8]. However, there are few studies on the relationship between DC-SIGN and TLRs in teleost fish, which needs further study.

A C-type lectin homologous to DC-SIGN was identified from *M. salmoides*. The recombinant MsDC-SIGN-CRD protein (rCRD) was purified to explore its immune functions in vitro. Meanwhile, the expression of key genes in the DC-SIGN and TLR signaling pathways post *A. hydrophila* stimulation in vivo was investigated. Further studies were carried out to explore the relationship between the DC-SIGN and TLR signaling pathways with RNAi technology. This study can help us understand the immune function and mechanism of *M. salmoides* DC-SIGN.

## 2. Results

### 2.1. Sequence Characters and Multiple Alignments of MsDC-SIGN

**Figure 1:**
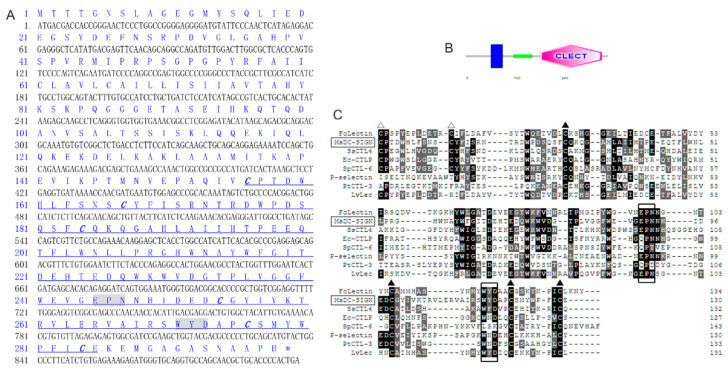
Molecular characteristics of MsDC-SIGN. (**A**) Nucleotide and deduced amino acid sequences of MsDC-SIGN. Nucleotides and amino acids are numbered. CRD domains are underlined and conserved cysteines are shown in bold italics. The motifs governing carbohydrate-binding specificity are shadowed. (**B**) The predicted structure domains of MsDC-SIGN by SMART. The blue region is the transmembrane region, the green region is coiled-coil, and the pink region is CRD. (**C**) ClustalW multiple sequence alignment of MsDC-SIGN with other C-type lectins, including FcLectin (AAX63905.1, *Penaeus chinensis*), SsCTL4 (AXQ05184.1, *S. schlegelii*), Ec-CTLP (AGM15882.1, *Epinephelus coioides*), SpCTL-6 (AIC80997.1, *Scylla paramamosain*), P-selectin (NP_002996, *H. sapiens*), PtCTL-3 (ATE51203.1, *Portunus trituberculatus*), LvLec (ABU62825.1, *Penaeus vannamei*), and MsDC-SIGN (XP_038580390.1, *Micropterus salmoides*). Amino acid residues that are conserved in at least 50% of sequences are shaded dark gray, and similar amino acids are shaded light gray. Conserved cysteine residues involved in the formation of CRD internal disulfide bridges are marked with ▲, and the two extra cysteine residues in the long-form are marked with △. The motifs determining the carbohydrate-binding specificity are boxed.

The protein-encoding sequence (CDS) of MsDC-SIGN spans 897 bp in length, and the open reading frame (ORF) encodes 298 amino acids. Based on the obtained protein sequence, the molecular weight (MV) of the protein was predicted to be 33.2 kilodaltons (kDa), and the theoretical isoelectric point (pI) of the protein was calculated to be 5.47. The polypeptide was predicted using SMART, revealing that the MsDC-SIGN molecule encompasses a transmembrane region, a helical region, and a carbohydrate recognition domain (CRD). The transmembrane region commences at the 54th amino acid and concludes at the 76th amino acid, totaling 23 amino acids. The helical region begins at the 100th amino acid and terminates at the 139th amino acid, while the CRD starts at the 156th amino acid and extends until the 285th amino acid (Figure 1A,B).

To analyze the sequence characteristics of the CRDs in MsDC-SIGN (Figure 1C), a multiple sequence alignment of the CRDs of DC-SIGN was conducted. In the extracellular segment of MsDC-SIGN’s CRD, it contains an EPN motif (Glu-Pro-Asn), which serves as a mannose-binding site, as well as a WYD motif (Trp-Tyr-Asp) and six conserved cysteines.

### 2.2. Recombinant Protein of CRD

**Figure 2:**
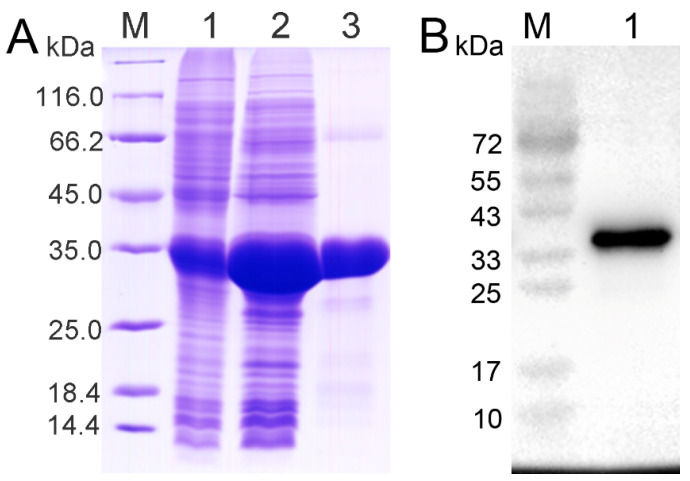
Analysis of rCRD by SDS-PAGE (**A**) and Western blot (**B**). (**A**) Lane M: protein molecular standard; Lane 1: negative control of rCRD (without induction); Lane 2: cell lysate of rCRD with induction; Lane 3: purified rCRD. (**B**) Lane M: prestained protein molecular standard; Lane 1: Western blot based on the purified rCRD of MsDC-SIGN with mouse serum antibody IgG.

The supernatant was collected by centrifugation containing rCRD, and purified rCRD was analyzed by SDS-PAGE. Distinct bands with a molecular weight of 34 kDa were observed, which was consistent with the predicted molecular weight (Figure 2A). The specificity of the polyclonal antibody of rCRD was detected by Western blotting. The experimental results showed that the ECL imaging results were consistent with the band size and position in the purified SDS-PAGE electrophoresis, and the antibody could be used in subsequent experiments (Figure 2B).

### 2.3. PAMP Binding Activity of MsDC-SIGN

**Figure 3:**
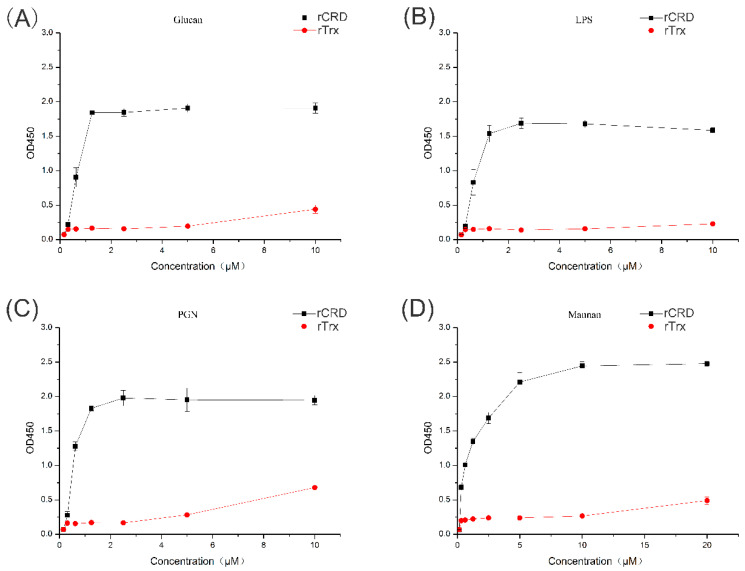
ELISA investigation of the interaction between rCRD and PAMPs. Plates were coated with four PAMPs and then incubated with rCRD and rTrx at different concentrations. After incubation with the rabbit anti-6×His-tag (HRP) polyclonal antibody (1:4000, Abcam), interactions between rCRD and PAMPs were detected following the instructions of the EL-TMB Chromogenic Reagent kit (Sangon) at 450 nm. (**A**) The binding curve of rCRD to glucan; (**B**) the binding curve of rCRD to LPS; (**C**) the binding curve of rCRD to PGN; (**D**) the binding curve of rCRD to mannan.

The binding activity of rCRD to various PAMPs was detected by ELISA. rCRD showed binding activity to all four PAMPs tested, including glucan, LPS, PGN, and mannan. The binding to PAMPs was dose-dependent, in which the binding affinity to mannan was the strongest (Figure 3). No binding affinity was observed for rTrx controls.

### 2.4. Microbe-Binding Activity of rCRD

**Figure 4:**
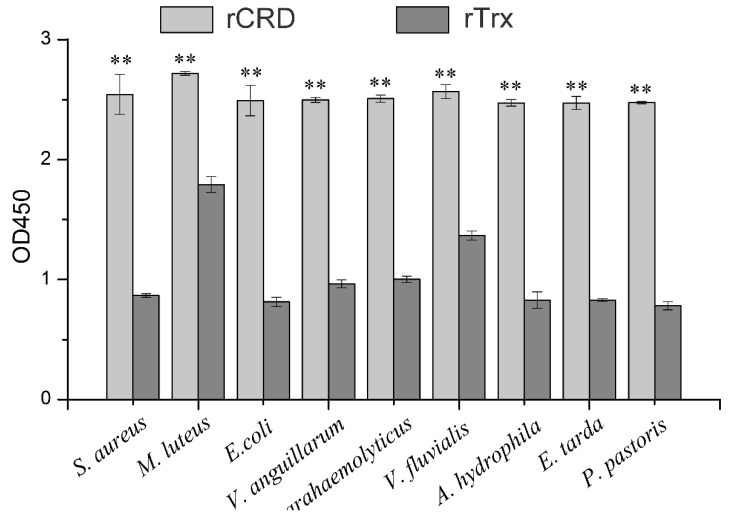
The microbe-binding spectrum of rCRD assessed by ELISA. Plates coated with microbes were incubated with rCRD and rTrx. After incubation with the rabbit anti-6×His-tag (HRP) polyclonal antibody (1:4000, Abcam), interactions between microbes and recombinant proteins were detected following the instructions of the EL-TMB Chromogenic Reagent kit (Sangon) at 450 nm. ** indicates an extremely significant difference (*p* < 0.01).

The binding activity of rCRD to various microorganisms was detected by ELISA. The experimental results showed that compared with the negative control Trx protein, rCRD had significant binding activity to all tested microorganisms, including two Gram-positive bacteria (*M. luteus* and *S. aureus*), six Gram-negative bacteria (*V. fluvialis*, *Aeromonas hydrophila*, *E. coli*, *V*. *parahaemolyticus*, *V. alginolyticus*, and *E. tarda*), and a fungus (*P. pastoris*) (Figure 4).

### 2.5. Microbial Binding Activity of rCRD

**Figure 5:**
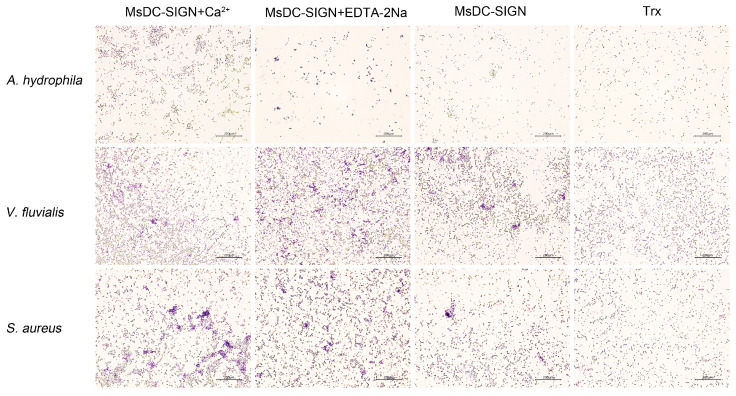
Microorganism agglutination activity of rCRD of MsDC-SIGN. Microorganisms (*A*. *hydrophila*, *V. fluvialis*, and *S*. *aureus*) were first washed with TBS buffer and then resuspended to a concentration of 1 × 10^8^ cells mL^−1^. They were then separately mixed with rCRD at a concentration of 10 μM, with rTrx used as a negative control. CaCl_2_ or EDTA-2Na was added to reach a final concentration of 5 mM, and the samples were incubated at 30 °C for 30 min. After incubation, the samples were fixed with 4% paraformaldehyde, clinical slices were prepared, stained with Giemsa stain, and observed under a light microscope to detect agglutination.

A. hydrophila, V. fluvialis, and S. aureus were utilized to assess the agglutination activity of the recombinant CRD of MsDC-SIGN. Regardless of the presence of calcium ions, rCRD exhibited agglutinating activity against all three bacteria (Figure 5), indicating that the agglutination was Ca^2+^-independent. No agglutination was observed in the control groups of rTrx.

### 2.6. Carbohydrate-Binding Specificity of rCRD

**Figure 6:**
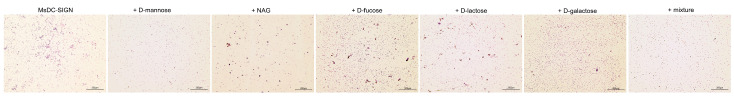
The carbohydrate-binding specificity of rCRD was determined by agglutination inhibition assay. rCRD was preincubated with D-mannose, N-acetylglucosamine (NAG), D-fucose, D-lactose, D-galactose, and mixtures of the aforementioned carbohydrates. Subsequently, rCRD, along with the carbohydrates, was incubated with *S. aureus*. The agglutination inhibition activity was observed.

Carbohydrates were used to preliminarily probe the binding specificity of rCRD of MsDC-SIGN. Upon preincubation with D-mannose, D-galactose, or a combination of five types of carbohydrates, the agglutinating activity of rCRD was suppressed. Nevertheless, preincubation with NAG, D-lactose, and D-fucose did not inhibit its agglutination activity, and S. aureus continued to agglutinate (Figure 6).

### 2.7. Subcellular Localization of MsDC-SIGN

**Figure 7:**
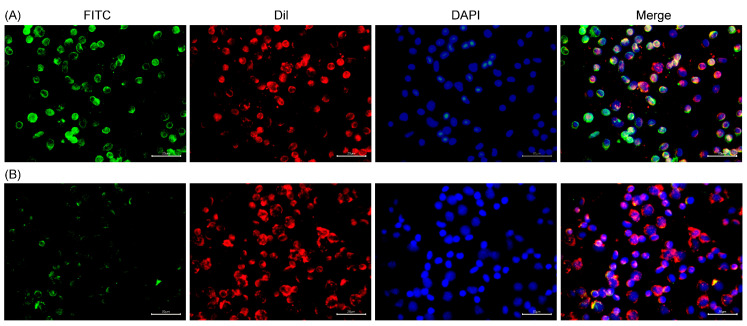
Confocal analysis of MsDC-SIGN distribution on leukocytes. Leukocytes were stained with polyclonal antibody against rCRD followed by Alexa Fluor 488-labeled anti-mouse immunoglobulin (IgG) antibody staining. Dil and DAPI were used to stain cell membrane and cell nucleus, respectively. (**A**) Leukocytes incubated with polyclonal antibody against rCRD; (**B**) leukocytes incubated with mouse negative control antibody.

Using the immunofluorescence method, the subcellular localization of the MsDC-SIGN protein was identified. MsDC-SIGN is mainly located on the cell membrane of *M. salmoides* leukocytes (Figure 7).

### 2.8. Phagocytosis Enhancement Activity of MsDC-SIGN

**Figure 8:**
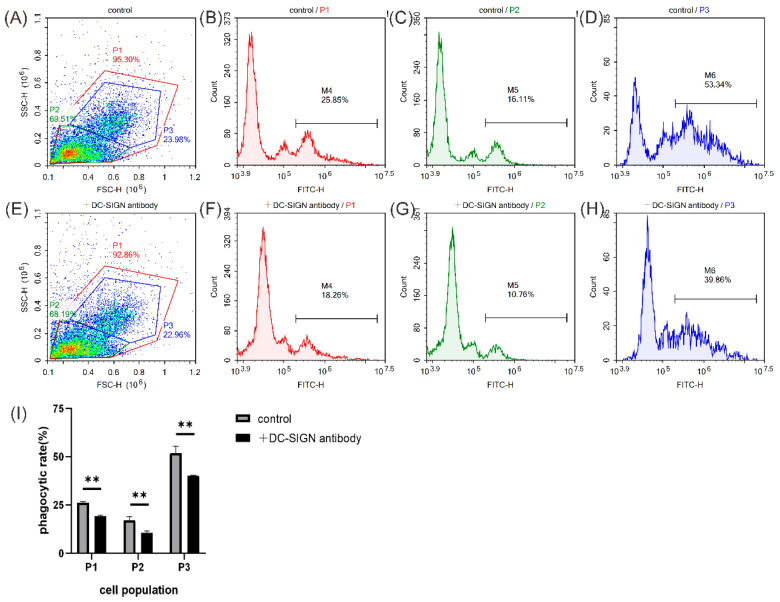
Flow cytometric analysis of phagocytosis of largemouth bass leukocytes to *A*. *hydrophila*. (**A**) Leukocytes in the peripheral blood were gated (P1, P2, and P3) on an FSC/SSC dot plot. (**B**–**D**) The fluorescence histogram showing the percentage of phagocytic leukocytes gated in P1, P2, and P3. (**E**) Leukocytes in the peripheral blood incubated with the antibody against rCRD were gated (P1, P2, and P3) on an FSC/SSC dot plot. (**F**–**H**) The fluorescence histogram showing the percentage of phagocytic leukocytes blocked with the antibody against rCRD gated in P1, P2, and P3. (**I**) Statistical analysis of the phagocytosis rate of leukocytes without or with the antibody against rCRD blocking. The asterisk on the bars represents statistical significance of phagocytic rates between two groups (** *p* < 0.01).

To investigate the phagocytosis enhancement activity of MsDC-SIGN, the antibody of rCRD was incubated with leukocytes in vitro. Flow cytometric analysis showed that the phagocytic rate of leukocytes gated in P1, P2, and P3 (Figure 8A,E) to *A*. *hydrophila* was calculated to be 19.07%, 10.69%, and 40.18% in the rCRD antibody-treated group (Figure 8F–H), which was significantly lower than that of the control group (26.12%, 17.08%, and 51.91%) (Figure 8B–D). The results showed that the antibody of rCRD significantly inhibited the phagocytosis activity of leukocytes gated in P1–P3 to *A*. *hydrophila* (Figure 8I).

### 2.9. Tissue Distribution of Key Genes in MsDC-SIGN and TLR Signaling Pathways

**Figure 9:**
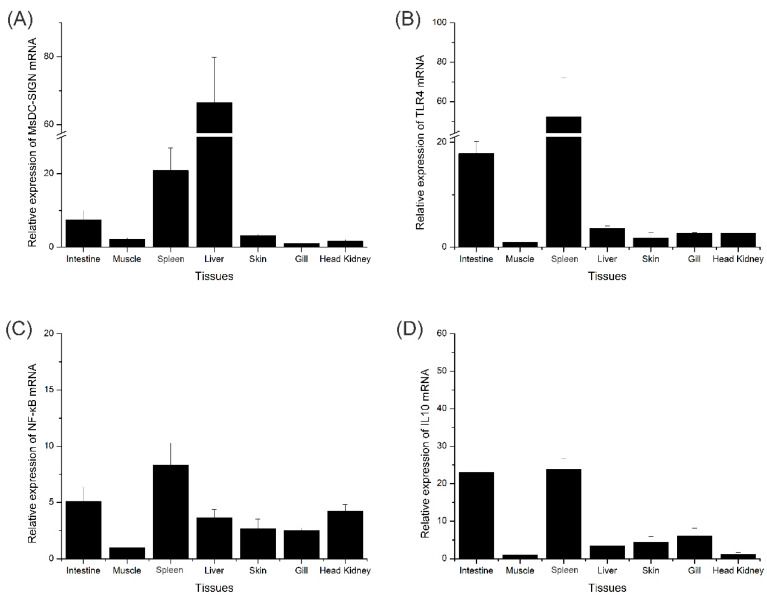
The tissue distribution pattern of MsDC-SIGN, TLR4, NF-κB, and IL10. (**A**) The tissue distribution pattern of MsDC-SIGN; (**B**) the tissue distribution pattern of TLR4; (**C**) the tissue distribution pattern of NF-κB; (**D**) the tissue distribution pattern of IL10. The β-actin gene was used as the internal control. The results are presented as the mean ± S.E. (n = 5).

The relative expression levels of MsDC-SIGN and three genes, namely, IL10, TLR4, and NF-κB, in various tissues of *M*. *salmoides* were acquired via RT-PCR (Figure 9). MsDC-SIGN exhibits significant expression in the liver, spleen, and intestine (Figure 9A). TLR4 showcases prominent expression in the gills, intestine, spleen, and liver (Figure 9B). NF-κB manifests heightened expression in the intestine, spleen, liver, and head kidney (Figure 9C). IL10 presents notable expression in the intestine, gills, skin, and liver (Figure 9D). It is evident that the aforementioned genes are expressed in the intestine, spleen, and liver, with a relatively elevated expression level. Consequently, for the subsequent bacterial stimulation experiments, the sampling and analysis shall focus on the selection of intestinal and hepatic tissues.

### 2.10. Expression Patterns of Key Genes in M. salmoides’ C-Type Lectin MsDC-SIGN and TLR Signaling Pathways after Bacterial Stimulation

**Figure 10:**
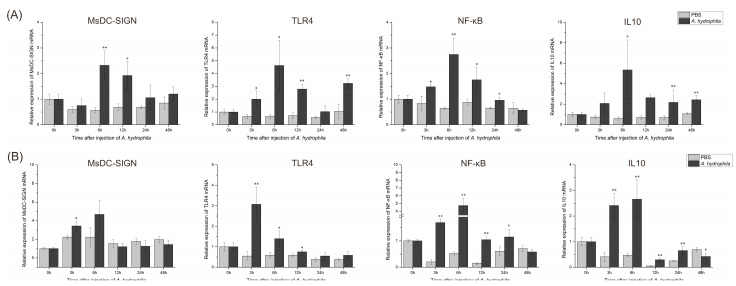
The expression levels of MsDC-SIGN and TLR4 signaling pathway genes post stimulation of *M. salmoides* by *A. hydrophila*. (**A**) The expression pattern of MsDC-SIGN, TLR4, NF-κB, and IL10 in the intestine post stimulation. (**B**) The expression pattern of MsDC-SIGN, TLR4, NF-κB, and IL10 in the liver post stimulation. * Indicates that there is a significant difference between the two groups of data in the experimental group and the control group (*p* < 0.05); ** indicates that the difference between the two groups of data in the experimental group and the control group is extremely significant (*p* < 0.01).

After stimulating *M. salmoides* with *A. hydrophila*, the expression levels of four genes, MsDC-SIGN, IL10, TLR4, and NF-κB, were analyzed over time in the tissues of the intestine and liver. The temporal changes in the four genes, MsDC-SIGN, IL10, TLR4, and NF-κB, in the intestine (Figure 10A) and liver (Figure 10B) were all gradually up-regulated from 3 or 6 h to 12 h post stimulation. Then, the upward trend of expression gradually weakened or decreased to a normal level at 24 or 48 h post stimulation (Figure 10).

### 2.11. Expression Patterns of Key Genes in M. salmoides’ TLR Signaling Pathway after RNA Interference with MsDC-SIGN Gene

**Figure 11:**
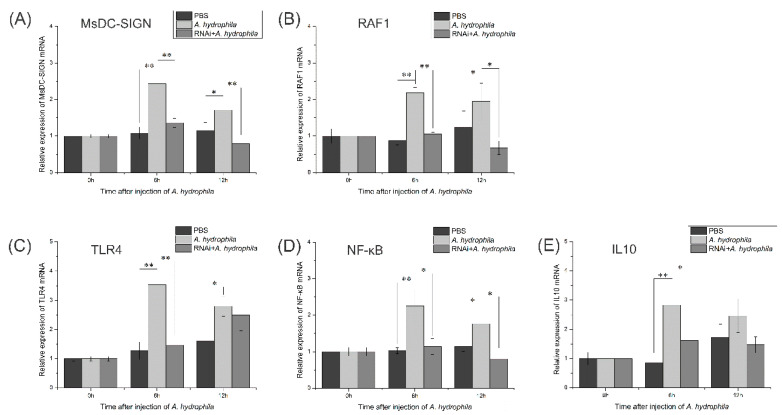
Expression levels of key genes in the DC-SIGN signaling pathway and the TLR signaling pathway after MsDC-SIGN silencing and *A. hydrophila* stimulation. (**A**) Expression level of the MsDC-SIGN gene. (**B**) Expression level of the RAF1 gene. (**C**) Expression level of the TLR4 gene. (**D**) Expression level of the NF-κB gene. (**E**) Expression level of the IL10 gene. * Indicates that there is a significant difference between the two groups of data in the experimental group and the control group (*p* < 0.05); ** indicates that the difference between the two groups of data in the experimental group and the control group is extremely significant (*p* < 0.01).

One set of siRNAs for RNA interference experiments was selected after screening (Supplementary Data, Figure S1). Following the successful silencing of the MsDC-SIGN gene through RNA interference, *M*. *salmoides* was subjected to stimulation with *A*. *hydrophila*. In comparison to the group that did not undergo RNA interference, the expression levels of these five genes exhibited a remarkable decline after 6 h of stimulation (Figure 11A–E). As the duration of infection prolonged, the expression levels of MsDC-SIGN, RAF1, and IL10 remained significantly down-regulated even after 12 h of stimulation in contrast to the group that did not undergo RNA interference.

## 3. Materials and Methods

### 3.1. M. salmoides and Microbes

Firstly, the mature *M*. *salmoides* for the experiment were acquired from a marketplace in Hangzhou. The mean weight was 500 ± 10 g, while the body length averaged 23 ± 2 cm. Secondly, the juvenile *M. salmoides* were procured from a pisciculture facility in Chongqing. The juvenile *M*. *salmoides* exhibited an average weight of 1 ± 0.2 g and a body length of 5 ± 1 cm. Subsequently, the procured juveniles and mature *M*. *salmoides* were harbored in an aquarium within a piscatory laboratory for a week, ensuring their sound state of well-being, prior to their utilization as experimental specimens.

*Micrococcus luteus* and *Escherichia coli* were purchased from the Microbial Culture Collection Center (Beijing, China). *V. parahaemolyticus*, *Vibrio fluvialis,* and *Vibrio alginolyticus* were purchased from the Global Biological Resource Center (United States). *Pichia pastoris* GS115 was from Invitrogen. *A. hydrophila* and *Edwardsiella tarda* were preserved by our laboratory.

### 3.2. Cloning and Sequence Analysis of Full-Length cDNA

The sequence of Ms-DC-SIGN was retrieved from the *M. salmoides* transcriptome database in our laboratory and it was blasted to the NCBI database (http://www.ncbi.nlm.nih.gov/blast, accessed on 1 September 2021). Then, the cDNA sequence was analyzed by using the Expert Protein Analysis System (http://www.expasy.org, accessed on 1 September 2021), the Simple Modular Architecture Research Tool (SMART) 7.0 (http://smart.embl-heidelberg.de/, accessed on 1 September 2021), and the SignalP 5.0 server (https://services.healthtech.dtu.dk/service.php?SignalP-5.0, accessed on 1 September 2021). The ClustalW Multiple Alignment software (http://www.ebi.ac.uk/clustalw/, accessed on 1 September 2021) was utilized to generate the multiple-sequence alignment.

### 3.3. Expression and Purification of rCRD

The cDNA sequence of the CRD in MsDC-SIGN was recombined to the pET-30a (+) vector and transformed into *E. coli* BL21 (DE3) competent cells. The recombinant strains of CRD were cultured in Luria broth (LB) medium containing 100 μg/mL of kanamycin at 37 ℃ until the absorbance at 600 nm (OD600) reached 0.4–0.6. Then, isopropyl β-D-1-thiogalactopyranopyranoside (IPTG) with a final concentration of 0.01 mM was added to the cultivation system. The recombinant strain of the CRD strain was incubated at 16 ℃ with shaking at 180 rpm for another 16 h. The bacteria were harvested after cultivation, resuspended in TBS buffer, and ultrasonicated. The CRD-soluble protein extracted from the supernatant was purified through Ni^2+^ chelating and Sepharose column chromatography, subsequently separated via reducing 12% sodium dodecyl sulfate–polyacrylamide gel electrophoresis (SDS-PAGE), and visualized through staining with Coomassie Brilliant Blue R250.

### 3.4. The PAMP Binding Assay

Various PAMPs were individually dissolved in a sodium carbonate–sodium bicarbonate buffer with a concentration of 50 mM. The 96-well plates were coated overnight at 4 °C with 10 μg of PAMPs per well. Subsequently, each well was supplemented with 200 μL of blocking buffer containing 3% BSA and incubated at 37 °C for 1 h. Following three rinses with a phosphate-buffered saline–Tween (PBS-T) solution at a pH of 7.4, each well received 200 μL of rCRD protein with 5 mM of CaCl_2_ through a double-gradient dilution method. The incubation took place at 18 °C for 3 h. Negative controls were established using rTrx at an equivalent concentration. After three PBS-T rinses, 100 μL of rabbit anti-6×His tag (HRP) polyclonal antibody (diluted to 1:4000, Abcam) was added to each well and incubated at 37 °C for 1 h. The results were assessed according to the instructions provided by the TMB Chromogenic Kit (Sangon), and the absorbance at 450 nm was measured using a microplate reader. For each experiment, three sets of replicates were arranged. The binding curves of rCRD were graphed utilizing Prism 7.00 software (GraphPad software).

### 3.5. Microbe Binding Assay

In accordance with the methodology elucidated by Wang [12], Gram-positive microorganisms (*S*. *aureus* and *M*. *luteus*), Gram-negative microorganisms (*E*. *coli*, *V*. *fluvialis*, *V*. *alginolyticus*, *V*. *parahaemolyticus*, *A*. *hydrophila*, and *E*. *tarda*), as well as the fungus *P*. *pastoris* were employed to scrutinize the microbial affinity of rCRD. Following centrifugation and collection of the cultivated microorganisms, they were reconstituted in a solution containing 50 mM of sodium carbonate–sodium bicarbonate. Subsequently, the bacterial suspension was dispensed into individual wells of a 96-well microtiter plate, with a volume of 100 μL per well, and left to incubate overnight at a temperature of 4 °C. Subsequent to being subjected to three rounds of rinsing using PBS-T, a solution of 3% BSA was introduced into each well to impede further binding and incubated at a temperature of 37 °C for a duration of 1 h. After being washed thrice again with PBS-T, rCRD was introduced into each well. The protein concentrations were adjusted to be uniform by employing a solution of 5 mM TBS-Ca^2+^, after which the plate was incubated at a temperature of 18 °C for a duration of 3 h. The negative control consisted of Trx. Following this, the plate was washed thrice using PBS-T. An aliquot of 100 μL of rabbit anti-6×His tag (HRP) polyclonal antibody (diluted to a ratio of 1:4000, Abcam) was added into each well and incubated at a temperature of 37 °C for a duration of 1 h. The ensuing outcomes were ascertained and evaluated using the same aforementioned methodology as in Section 2.5.

### 3.6. Microbial Agglutination and Ca^2+^-Dependent Assay

Microorganisms (*A*. *hydrophila*, *V. fluvialis*, and *S*. *aureus*) were rinsed with TBS buffer and reconstituted to a concentration of 1 × 10^8^ cells mL^−1^. They were then separately combined with rCRD at a concentration of 10 μM (or rTrx as a negative control). CaCl_2_ was introduced to achieve a final concentration of 5 mM, and the samples were incubated at 30 °C for 30 min. The experiments were replicated using TBS or TBS-EDTA buffer (TBS buffer containing 5 mM EDTA-2Na, pH 7.5) under the same conditions to ascertain whether the agglutination of microbes necessitated Ca^2+^. Following this, the samples were fixed with 4% paraformaldehyde, clinical slices were prepared and stained with Giemsa stain, and agglutination was observed under a light microscope.

### 3.7. Carbohydrate-Binding Specificity Assay

The carbohydrate-binding specificity of rCRD was determined using the aforementioned method, albeit with certain modifications. *S. aureus* cells were washed with TBS buffer and adjusted to a concentration of 1 × 10^8^ cells⋅mL^−1^. Subsequently, they were individually mixed with rCRD (10 μM) in the presence or absence of various carbohydrates (D-mannose, N-acetylglucosamine (NAG), D-fucose, D-lactose, and D-galactose) at a final concentration of 250 mM. Additionally, a mixture of the five carbohydrates was incubated with rCRD, and the resulting samples were then incubated at 30 °C for 30 min. The agglutination inhibition activity was observed using a light microscope.

### 3.8. Preparation and Western Blot Identification of rCRD Polyclonal Antibody

rCRD was suspended in phosphate-buffered saline (PBS) (140 mM NaCl, 2.7 mM KCl, 10 mM Na_2_HPO_4_, and 1.8 mM KH_2_PO4, pH 7.4), and injected into 6-week-old mice to generate polyclonal antibodies, following the procedure outlined by Pan et al. [13]. The IgG of the rCRD antibody was purified using protein A agarose (Beyotime, China). The specificity of the antibody was detected by Western blot analysis [14].

### 3.9. Immunofluorescence Analysis of the Subcellular Localization of MsDC-SIGN

Immunofluorescence analysis was carried out as described in Li. et al., with some modifications [15]. *M. salmoides* leukocytes were extracted with Percoll reagent, and the number of leukocytes were adjusted to 2 × 10^6^ CFU/mL with PBS buffer. An appropriate number of leukocytes were dropped onto the adhesive glass slide, and the wet box settled for 30 min. Cells were fixed on glass slides with 4% paraformaldehyde for 1 h. Slides were washed with PBS solution. An appropriate amount of 0.2% Triton X-100 solution was added dropwise to the glass slide to permeabilize the cells for 5 min. After washing the slides with PBS solution, an appropriate amount of 3% BSA solution was added and blocked for 1 h at room temperature. After washing the slides with PBS solution, the rCRD antibody was added and incubated overnight at 4 °C. After washing the slides with PBS solution, anti-mouse fluorescently labeled secondary antibody Alexa Fluor 488 was added and incubated at room temperature for 1 h. After washing the slides with PBS solution, they were stained with DAPI working solution for 10–15 min. A laser scanning confocal microscope was used to observe the slides and take pictures to store the results. The mouse negative antibody was used as a control group.

### 3.10. Phagocytosis Assay of Leukocytes

*A*. *hydrophila* was cultured in LB medium at 37 °C and collected in the logarithmic growth phase. The collected bacteria were washed three times with 0.1 mM sodium bicarbonate solution and incubated with 4% paraformaldehyde at 25 °C for 2 h to inactivate the bacteria. The inactivated *A*. *hydrophila* were labeled with 0.1 mg/mL fluorescein isothiocyanate (FITC) at 25 °C for 2 h, washed with PBS five times to remove excess FITC, and finally resuspended to 1.0 × 10^8^ CFU/mL for use.

The leukocytes were isolated from largemouth bass by Percoll density gradient. The cells were resuspended with 1640 medium and the cell concentration was adjusted to 1.0 × 10^6^ CFU/mL. Then, 500 μL of leukocyte suspension was incubated with 5 μL anti-rCRD antibody or mouse negative serum for 1 h at 25 °C, respectively. Then, 50 μL of FITC-labeled *A*. *hydrophila* was added and the mixtures were incubated at 25 °C for 30 min. Following incubation, the cell mixtures were laid over 3 mL PBS containing 3% BSA and 4.5% D-glucose and centrifuged at 100 g at 4 °C for 10 min to remove unengulfed *A*. *hydrophila*. Finally, the leukocytes were washed three times with PBS and detected using a NovoCyte Advanteon Flow Cytometer (Agilent, Santa Clara, CA, USA).

### 3.11. RNA Isolation and cDNA Synthesis

The intestine, muscle, spleen, liver, skin, gills, and head kidney of *M. salmoides* were sampled from a total of seven tissues, and five fish were taken from each tissue as five replicates. Immediately after sampling, the tissue samples were placed in liquid nitrogen for freezing, and immediately after the quick freezing, the tissues were placed in a −80 °C refrigerator for future use.

The RNA of *M. salmoides* tissues were extracted according to the method of Pan. et al. with some modifications [16]. The cDNA was synthesized using the FastKing cDNA First Strand Synthesis Kit (TIANGEN, Beijing, China).

### 3.12. Tissue Distribution of Key Genes in MsDC-SIGN and TLR Signaling Pathways

Primer Premier 5.0 software was used to design primers for qRT-PCR, and the primer sequences used are shown in Table 1. The key genes IL10, TLR4, and NF-κB of the MsDC-SIGN and TLR signaling pathways were selected, and the expression levels of these genes in various tissues of *M. salmoides* were analyzed by conducting qRT-PCR experiments. Each cDNA tissue template of *M. salmoides* was prepared using the Real-Time PCR EasyTM-SYBR Green I kit (Foregene, Chengdu, China).

### 3.13. Expression Patterns of Key Genes in M. salmoides’ C-Type Lectin MsDC-SIGN and TLR Signaling Pathway after Bacterial Stimulation

Three tissues with higher expression levels were selected, namely, the intestine, liver, and spleen were used for experiments. *A. hydrophila* was used to stimulate *M. salmoides* juveniles. Six time points after stimulation were selected, three tissue samples were collected, and cDNA templates were prepared. qRT-PCR technology was used to analyze the changes in the expression levels of each gene over time.

### 3.14. Expression Patterns of Key Genes in M. salmoides’ TLR Signaling Pathway after RNA Interference with MsDC-SIGN Gene

The RNAi was performed according to the method mentioned by Ye. et al., with some modifications [17]. Pairs of potential siRNAs against *M. salmoides* MsDC-SIGN mRNA were designed using two online programs (http://biodev.extra.cea.fr/DSIR/DSIR.htmL and http://sidirect2.rnai.jp/, accessed on 1 September 2021). In addition to this, a non-targeting sequence (NC siRNA) was synthesized as a negative control. The RNA interference primer sequences used are shown in Table 2.

The MsDC-SIGN siRNA was introduced into the fish body through intraperitoneal injection, followed by the simultaneous injection of *A*. *hydrophila* to induce an immune response in the juvenile *M*. *salmoides*. Subsequently, intestinal tissue samples from each group of fish were collected for analysis. The key gene RAF1 of the DC-SIGN signaling pathway and the key genes IL10, TLR4, and NF-κB of the TLR signaling pathway were selected to detect their expression mode.

### 3.15. Data Statistics and Analysis

The data obtained by using the qRT-PCR technology were analyzed, and each group of data was the average of five parallel experiments. The experimental data were processed using the calculation method of 2^−ΔΔCT^ [18]. Significant differences among groups were tested by one-way analysis of variance and multiple comparisons. Statically significant differences were designated at *p* < 0.05, and extremely significant differences at *p* < 0.01. OriginLab Origin 8.1 software was used to draw statistical graphs.

## 4. Discussion

C-type lectins play an important role in the identification and removal of invaders, which has attracted more and more researchers’ attention. In the present study, a C-type lectin was identified from *M. salmoides,* which was identified as a DC-SIGN molecular protein, with the key motif EPN/WYD in Ca^2+^-binding site 2. Studies have shown that in the CRD of C-type lectins, there are four or six conserved Cys, which can form two or three pairs of disulfide bonds, and they can be used to build and stabilize the “double ring” structure of CRD [19]. Employing multiple-sequence alignment, it was ascertained that MsDC-SIGN possesses six Cys within its CRD structure, with four cysteine residues establishing two pairs of conserved disulfide bonds. Notably, a pair of disulfide bonds is formed by two Cys situated at the N-terminal of the polypeptide, hence conferring upon the CRD of MsDC-SIGN the designation of a prototypical ‘long form’ CRD.

C-type lectins can recognize and bind to various PAMPs and trigger a series of immune responses, thereby activating the host’s defense system [20]. There are two kinds of key motifs in Ca^2+^-binding site 2 for mammal C-type lectins, which are EPN/WND and QPD/WND. C-type lectins with the EPN/WND motif can specifically bind to D-mannose, while C-type lectins with QPD/WND can specifically bind to D-galactose [21,22]. The DC-SIGN molecule contains a mannose-binding site EPN, which can recognize carbohydrates containing mannose. However, the WND motif in the CRD of MsDC-SIGN became WYD. Different motifs of C-type lectins can cause differences in their sugar-binding specificities [23]. Studies on human DC-SIGN have found that DC-SIGN can bind to mannose-containing carbohydrates [24]. In related studies on fish, the C-type lectin CaNTC from *Carassius auratus* and rOmCTL and rOmLec1 from *Onychostoma macrolepis* all contain EPN/WND motifs and have certain binding activities to LPS and PGN [25,26]. The C-type lectin SsCTL4 from *S. schlegelii* contained an EPN/WFD (Trp-Phe-Asp) motif, which could bind to LPS and PGN [8]. In the current investigation, MsDC-SIGN exhibited dose-dependent binding to LPS, glucan, PGN, and mannan, with the most pronounced affinity for mannan, indicating a specificity for mannose binding. However, the agglutination inhibition assay showed that rCRD can be inhibited not only by D-mannose, but also by D-galactose. This phenomenon is commonly observed in the C-type lectins of invertebrates [27], indicating that as lower vertebrates, the sugar-binding specificity of C-type lectins in teleost differs from that in higher vertebrates, requiring further investigation.

C-type lectins can bind to a variety of pathogenic microorganisms by recognizing microorganism-specific PAMPs, mediating agglutination against microorganisms [4]. In the studies of fish, the C-type lectin SsCTL4 from *S. schlegelii* has a significant binding affinity to *E. tarda* and *Vibrio anguillarum* and can agglutinate several kinds of bacteria [8]. The C-type lectin CaNTC from *C. auratus* can bind to *A. hydrophila*, *V. anguillarum*, *Streptococcus hemolyticus*, *E. coli*, *Bacillus subtilis,* and *S. aureus* and display agglutination activity toward microbes [26]. In this study, it was found that rCRD could bind to two Gram-positive bacteria (*M. luteus* and *S. aureus*), six Gram-negative bacteria (*Vibrio riviformis*, *A. hydrophila*, *E. coli*, *V. parahaemolyticus*, *V. alginolyticus*, and *E. tarda*), and one fungus (*P. pastoris*); their microbial binding profiles are consistent with the binding profiles of PAMPs. Moreover, rCRD could agglutinate Gram-positive and Gram-negative bacteria in an Ca^2+^-independent manner. 

Prior research has established that DC-SIGN is ubiquitously distributed on macrophages in the human placental tissue and on the surface of dendritic cells [24]. The results in teleosts were similar to those in human DC-SIGN studies. For instance, in zebrafish, DC-SIGN was identified to be expressed on macrophages, dendritic cells, and B lymphocytes [6]. This current investigation ascertained the presence of MsDC-SIGN on the cell membrane of leukocytes in *M*. *salmoides*, which corresponded to earlier discoveries.

Phagocytosis plays an important role in innate immune defense and the activation of adaptive immunity. Recently, several C-type lectins found in vertebrates, such as DC-SIGN, Dectin-1, and the mannose receptor, have been discovered to incite phagocytosis through interaction with microorganisms [24]. For example, human DCs expressing CD209 bound to LPS and promoted phagocytosis of *Yersinia pestis* [28]. In the teleost fish tongue sole, leukocytes incubated with CD209 significantly enhanced the phagocytosis of bacteria [29]. In the case of largemouth bass, we found that preincubation of the polyclonal antibody of MsDC-SIGN CRD with leukocytes significantly inhibited the phagocytosis of *A*. *hydrophila*, suggesting that MsDC-SIGN promoted the intracellular uptake of bacteria. Therefore, when coupled with the in vivo findings of MsDC-SIGN up-regulation in response to *A*. *hydrophila* stimulation, this indicates that MsDC-SIGN functions as an innate immune factor that contributes to the elimination of microbial pathogens.

The in vivo experiments and the analysis of qRT-PCR experimental data found that MsDC-SIGN and key genes of the TLR signaling pathway (TLR4, NF-κB, and IL10) were found in the intestine, muscle, spleen, liver, skin, gills and head kidney of *M. salmoides*. The expression of MsDC-SIGN was highest in the liver, while also exhibiting a significant level of expression in the spleen and intestine. Furthermore, within the TLR signaling pathway, the three key genes (TLR4, NF-κB, and IL10) were ubiquitously present in all examined tissues, with the spleen and intestine displaying a heightened expression level. Research has ascertained that the spleen possesses the capability to apprehend antigens [30], thereby playing a critical role in antigen presentation and initiating the innate immune system [31]. Studies have found that the spleen can capture antigens, which plays a vital role in antigen presentation and initiates the innate immunity system. Moreover, lymphocytes are present within the intestine epithelium of teleost fish, and B lymphocytes are present in the lamina propria in the intestine [32,33]. It can be inferred that the spleen and intestine in fish serve as organs that actively synthesize DC-SIGN and TLR-4 signaling pathway genes, thus assuming a pivotal role in pathogen elimination.

PRRs are expressed on effector cells of the innate immune system, which are the first to encounter non-self-invaders and to directly induce innate effector mechanisms or trigger the expression of endogenous signals such as inflammatory cytokines [34]. In order to further investigate the physiological function of MsDC-SIGN in *M*. *salmoides*, the analysis of expression patterns after stimulation with *A*. *hydrophila* was conducted. In the study of zebrafish, it was discovered that DC-SIGN expression was up-regulated in the spleen and gills following stimulation by *A*. *hydrophila* [6]. The relevant research on TLR4 revealed that the mRNA expression level of the Tf_TLR4 gene was up-regulated in the intestine and immune-related tissues of *Tachysurus fulvidraco* post *E. ictalurid* infection [35]. Subsequent studies found that DC-SIGN can impact TLR4-mediated immune responses following mycobacteria stimulation, suggesting that DC-SIGN can regulate the TLR4 signaling pathway [36]. In our investigation, we observed that both MsDC-SIGN and TLR4 can promptly up-regulate in response to *A*. *hydrophila* stimulation. After silencing MsDC-SIGN, the expression level of the RAF1 gene was significantly down-regulated. Simultaneously, the expression levels of key genes (TLR4, NF-κB, and IL10) in the TLR signaling pathway were also down-regulated. Hence, it is speculated that DC-SIGN can participate in the body’s elimination of invading pathogenic microorganisms by modulating the TLR signaling pathway in largemouth bass.

In conclusion, we identified and characterized MsDC-SIGN from largemouth bass, and assessed functional activities including non-self-recognition ability, phagocytosis enhancement activity, and the regulation effect on the TLR signaling pathway. This study showed that MsDC-SIGN, as a PRR, recognized and bound to PAMPs and microbes. Furthermore, it actively promoted the engulfment of bacteria by leukocytes through the potentiation of phagocytic activity. Upon stimulation of M. salmoides by A. hydrophila, the DC-SIGN signaling pathway effectively governed the TLR signaling pathway, facilitating the eradication of the invasive pathogens afflicting *M. salmoides*.

## Figures and Tables

**Table 1:** qRT-PCR primer sequences.

Primer Name	Primer Sequence (5′–3′)
β-actin-F	CCACCACAGCCGAGAGGGAA
β-actin-R	TCATGGTGGATGGGGCCAGG
MsDC-SIGN-F	ACGCCTACTGGTTTGGAATCAC
MsDC-SIGN-R	ATGTAGCCACAGTCCTCGTCAAT
RAF1-F	TCTACCTCCCAAACCAGCA
RAF1-R	CAGTGTTCCAATCCATCCG
IL10-F	AAGCCAGCAGCATCATTACCACT
IL10-R	AGAACCAGGACGGACAGGAGG
TLR4-F	TGATGCTTCTTGCTGGCTGC
TLR4-R	CAATCACCTTTCGGCTTTTATGG
NF-κB2-F	TGGCTGCCGAAACCGCT
NF-κB2-R	GCTGGACGAGGACACGCTG

**Table 2:** The RNA interference primer sequences.

Gene Name	Sequence	
	Sense (5’–3’)	Antisense (5’–3’)
MsDC-SIGN-siRNA	GUUUCUGUGGAAUCUUCUACC	UAGAAGAUUCCACAGAAACGU
NC	UUCUCCGAACGUGUCACGUTT	ACGUGACACGUUCGGAGAATT

## Data Availability

All datasets generated for this study are included in the article. The data that support the findings of this study but not presented in the figures, tables, and supplementary files are available upon reasonable request.

## Supplementary Materials

The following supporting information can be downloaded at: https://www.mdpi.com/article/10.3390/ijms25095013/s1.
