# Supplementary material for: DC-SIGN of Largemouth Bass (Micropterus salmoides) Mediates Immune Functions against Aeromonas hydrophila through Collaboration with the TLR Signaling Pathway
Source: Int J Mol Sci. 2024 May 3;25(9):5013. doi: 10.3390/ijms25095013 (PMC11084180; doi:10.3390/ijms25095013)
Supplement: Supplementary file 1 [file ijms-25-05013-s001.zip › ijms-2952566-supplementary.pdf]

The RNAi effects of different siRNAs for interference of MsDC-SIGN with different dose (Figure S1).

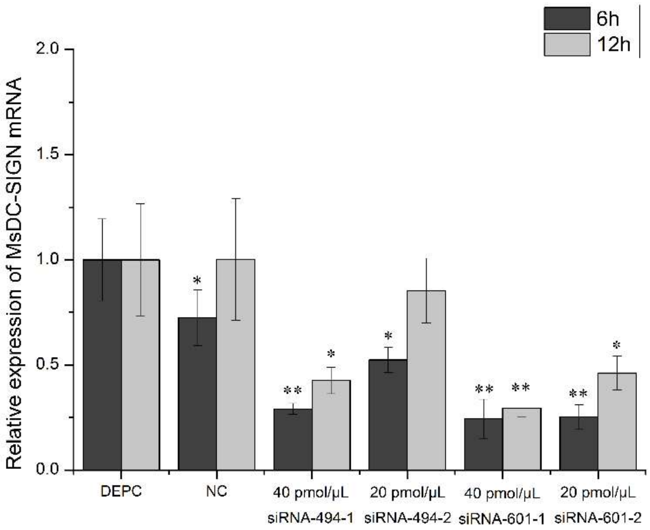

Figure S1. Expression level of MsDC-SIGN gene after RNA interference at 6 h and 12 h. * indicates that there is a significant difference between the two groups of data in the experimental group and the control group ($P < 0.05$); ** indicates that the difference between the two groups of data in the experimental group and the control group is extremely significant ($P < 0.01$).
